# Telehealth Brief Cognitive Behavioral Therapy for Suicide Prevention: A Randomized Clinical Trial

**DOI:** 10.1001/jamanetworkopen.2024.45913

**Published:** 2024-11-12

**Authors:** Justin C. Baker, Austin Starkey, Ennio Ammendola, Christina Rose Bauder, Samantha E. Daruwala, Jaryd Hiser, Lauren R. Khazem, Keelin Rademacher, Jarrod Hay, AnnaBelle O. Bryan, Craig J. Bryan

**Affiliations:** 1 Department of Psychiatry and Behavioral Health, The Ohio State University Wexner Medical Center, Columbus; 2 Department of Psychology, Louisiana State University, Baton Rouge

## Abstract

**Importance:**

Suicide rates continue to increase in the US. Evidence-based treatments for suicide risk exist, but their effectiveness when delivered via telehealth remains unknown.

**Objective:**

To test the efficacy of brief cognitive behavioral therapy (BCBT) for reducing suicide attempts and suicidal ideation among high-risk adults when delivered via telehealth.

**Design, Setting, and Participants:**

This 2-group parallel randomized clinical trial comparing BCBT with present-centered therapy (PCT) was conducted from April 2021 to September 2023 with 1-year follow-up at an outpatient psychiatry and behavioral health clinic located in the midwestern US. Participants reporting suicidal ideation during the past week and/or suicidal behavior during the past month were recruited from clinic waiting lists, inpatient service, intermediate care, research match, and direct referrals from clinicians. A total of 768 participants were invited to participate, 112 were assessed for eligibility, and 98 were eligible and randomly assigned to a treatment condition. Data analysis was from April to September 2024.

**Interventions:**

Participants received either BCBT, an evidence-based suicide-focused treatment that teaches emotion regulation and reappraisal skills, or PCT, a goal-oriented treatment that helps participants identify adaptive responses to stressors. Participants were randomized using a computerized stratified randomization algorithm with 2 strata (sex and history of suicide attempts).

**Main Outcomes and Measures:**

The primary outcome was suicide attempts as measured by the Self-Injurious Thoughts and Behaviors Interview-Revised. The secondary outcome was severity of suicidal ideation as measured by the Scale for Suicide Ideation.

**Results:**

Participants included 96 adults (mean [SD] age, 31.8 [12.6] years; 64 female [66.7%] and 32 male [33.3%]), with 51 receiving BCBT and 45 receiving PCT. Of all participants, 85 (88.5%) completed at least 1 session. From baseline to 12 months, 12 participants receiving PCT (estimated percentage, 35.6%) made 56 suicide attempts and 11 participants receiving BCBT (estimated percentage, 30.0%) made 36 suicide attempts. Participants randomized to BCBT made significantly fewer suicide attempts than participants randomized to PCT (mean [range], 0.70 [0.00-8.00] attempts per participant vs 1.40 [0.00-10.00] per participant) and had a 41% reduced risk for suicide attempts (hazard ratio, 0.59; 95% CI, 0.36-0.96; *P* = .03). Severity of suicidal ideation significantly decreased in both treatments (*F*_4,330_ = 50.1; *P* < .001) but did not differ between groups (*F*_4,330_  = 0.2; *P* = .91).

**Conclusions and Relevance:**

The findings of this randomized clinical trial suggest that BCBT delivered via video telehealth is effective for reducing suicide attempts among adults with recent suicidal thoughts and/or behaviors.

**Trial Registration:**

ClinicalTrials.gov Identifier: NCT04844294

## Key Points

**Question:**

Can brief cognitive behavioral therapy (BCBT) for suicide prevention reduce suicide attempts and suicidal ideation when delivered remotely via video telehealth?

**Findings:**

Results of this randomized clinical trial of 96 US adults with recent suicidal ideation and/or suicidal behavior show that patients who received BCBT had significantly fewer suicide attempts during the 1-year follow-up vs patients who received present-centered therapy. Reductions in suicidal ideation occurred in both treatments with no significant differences between groups.

**Meaning:**

These findings suggest that BCBT delivered via video telehealth is effective for reducing the risk of suicide attempts among adults with recent suicidal ideation and/or suicidal behavior.

## Teaser

This randomized clinical trial analyzes associations of brief cognitive behavioral therapy delivered via telehealth with reduction of suicide attempts among adults with recent suicidal ideation or behavior.

## Introduction

Suicide remains a pressing public health concern. An estimated 703 000 people die by suicide each year worldwide.^1^ In the US, there were 49 449 suicides in 2022.^2^ Since 2000, the US suicide rate has increased more than 33%. Nonfatal suicide attempts and suicidal ideation are even more common, with 12 million adults experiencing suicidal ideation and 1.7 million attempting suicide in the US.^3^

Suicide-focused cognitive behavioral therapies (CBTs) are shown to significantly reduce suicidal thoughts and behaviors,^4,5^ with recent clinical trials finding reductions of 50% or more in suicide attempts when compared with treatment as usual.^6,7,8^ Brief CBT (BCBT) for suicide prevention^9^ is an evidence-based transdiagnostic psychotherapy that has been previously demonstrated to reduce suicide attempts among active duty soldiers,^6^ civilian adolescents and young adults,^7^ inpatient patients,^10^ and veterans.^11^ Emotion regulation and cognitive flexibility are 2 proposed mechanisms of action.^12^ BCBT directly targets suicidal behavior by teaching patients to effectively regulate their emotions and cognitively reappraise when presented with stressors. However, no studies have investigated the safety and effectiveness of BCBT delivered via telehealth.

Abundant evidence supports the safety and effectiveness of mental health services delivered via telehealth^13,14,15,16,17^; however, the effectiveness of suicide-focused treatments delivered via telehealth remains unknown^18^ partly due to concerns about the safety of telehealth for patients with elevated risk of suicide.^19,20^ With the onset of the COVID-19 pandemic, however, telehealth rapidly became the dominant method for mental health service delivery, increasing by over 1400% in less than 1 month.^21,22^ Although a postpandemic return to in-person services has occurred, telehealth services will likely continue to be common given their broad acceptability and feasibility in facilitating effective care while addressing historical barriers to care.^23^ Research investigating the safety and effectiveness of suicide-focused interventions delivered via telehealth is therefore warranted.

The present study entails a phase 2 randomized clinical trial (RCT) comparing BCBT for suicide prevention with present-centered therapy (PCT), an evidence-based active control that has been shown to reduce suicidal ideation^24,25^ and psychiatric symptom severity.^26^ PCT was selected as an active control because it does not target the proposed mechanisms of action within BCBT. We hypothesized that patients randomly assigned to telehealth BCBT would report reduced suicide attempts (hypothesis 1) and reduced suicidal ideation (hypothesis 2) during the 1-year follow-up when compared with patients receiving telehealth PCT.

## Methods

### Design

The study design was a 2-group parallel RCT. Written informed consent was obtained from all participants. Study procedures were approved by The Ohio State University Behavioral and Social Sciences institutional review board and adhere to the Consolidated Standards of Reporting Trials (CONSORT) reporting guideline (see the trial protocol in Supplement 1).

### Setting and Participants

From April 2021 to September 2023, participants accessing care at an outpatient psychiatry and behavioral health clinic located in the midwestern US either self-referred to participate or were referred from psychiatric inpatient, intermediate care, clinic waiting lists, and behavioral health clinicians. Research staff attended clinical team meetings to identify potential participants. Prospective participants could also self-refer via Research Match, a secure National Institutes of Health–sponsored volunteer registry that allows eligible participants to be connected to studies. Inclusion criteria were (1) 18 years of age or older, (2) a score of 5 or higher on the Scale for Suicide Ideation (SSI)^27^ and/or a suicide attempt within the past month as assessed by items adapted from the Self-Injurious Thoughts and Behaviors Interview-Revised (SITBI-R),^28^ (3) ability to understand and speak English, (4) ability to complete the informed consent process, (5) regular access to a stable internet connection, and (6) ownership of an internet-enabled communication device (eg, computer, tablet, or smartphone). Exclusion criteria were (1) substance use disorder requiring acute medical management, (2) imminent suicide risk warranting inpatient hospitalization at time of consent, and (3) impaired mental status that precludes the ability to provide informed consent (eg, intoxication, psychosis, or mania). Exclusion criteria were intentionally broad to maximize generalizability.

### Procedures

All study procedures were conducted remotely. Patients were referred to our research staff for eligibility determination if they screened positive for suicidal ideation or verbally reported recent suicidal thoughts and behaviors. Referred patients were contacted by a member of the research team to explain the purpose of the study, procedures, and possible risks and benefits. After consenting to be screened, potential participants completed an eligibility assessment. Eligible participants next completed a second informed consent process outlining details of the RCT. After consenting, participants completed the baseline assessment via self-report. All data were collected remotely via the Research Electronic Data Capture (REDCap) version 14.6.4, a secure, web-based software platform designed to support data capture for research studies,^29,30^ hosted at The Ohio State University. Demographic variables followed the US Office of Management and Budget *Revisions to the Standards for the Classification of Federal Data on Race and Ethnicity*.^31^ Following the baseline assessment, participants were randomized to either BCBT or PCT using a computerized stratified randomization algorithm with 2 strata: biological sex (male or female) and history of suicide attempts (no previous attempts, 1 previous attempt, or 2 or more previous attempts).

All participants attended an intake session to meet their therapist, discuss guidelines for telehealth, and complete a diagnostic interview using the Diagnostic Interview for Anxiety, Mood, and Obsessive-Compulsive Disorder and Related Neuropsychiatric Disorders.^32^ Participants completed follow-up assessments via REDCap at 3, 6, 9, and 12 months. Participants received a $50 Amazon gift card for each follow-up assessment.

### Treatments

Participants were randomly assigned to either BCBT or PCT. To minimize bias associated with expectancy effects, participants were not informed about their treatment group assignment. Both treatments included an intake session followed by 12 weekly outpatient individual sessions. The intake session for both therapies was 90 minutes with subsequent sessions approximately 60 minutes. All therapy sessions were conducted remotely via telehealth. The specific procedures and components for each treatment are summarized in the Table 1.

**Table 1.** Treatment Procedures Used in BCBT and PCT

Treatment procedure or component	BCBT	PCT
Suicide risk screening	X	X
Narrative assessment	X	NA
Crisis response plan or safety plan	X	X
Means safety counseling	X	X
Weekly monitoring of suicide risk	X	X
Psychiatric symptom management	X	X
Psychoeducation: suicide as symptom of mental illness	NA	X
Psychoeducation: suicide as a deficit in self-regulation	X	NA
Emotion regulation skills training	X	NA
Cognitive restructuring skills training	X	NA
Relapse prevention task	X	NA

Abbreviations: BCBT, brief cognitive behavioral therapy; NA, not applicable; PCT present-centered therapy; X, procedure or component included.

#### BCBT

BCBT is a time-limited psychotherapy that focuses on teaching emotion regulation and cognitive reappraisal skills. BCBT is structured in 3 phases. In phase 1 (5 sessions), the therapist conducts a detailed assessment of the patient’s most recent suicidal crisis, identifies patient-specific factors that contribute to and maintain suicidal behaviors, provides a cognitive-behavioral conceptualization, collaboratively develops a crisis response plan, and teaches emotion regulation skills such as relaxation and mindfulness. In phase 2 (5 sessions), the therapist applies cognitive strategies to identify and modify beliefs and assumptions that maintain vulnerability to suicidal behavior (eg, hopelessness or perceived burdensomeness). In phase 3 (2 sessions), patients complete an imagery rehearsal relapse prevention task during which patients imagine the circumstances and internal experiences (eg, thoughts, emotions, and physical responses) of a past suicidal crisis and hypothetical future crises, then visualize themselves using newly learned skills to successfully regulate their emotional response and use problem solving skills to resolve the crises. If participants are unable to successfully complete the relapse prevention task, skills are reviewed, and the task is repeated in additional sessions until successfully completed.

#### PCT

PCT is a time-limited psychotherapy that focuses on increasing adaptive responses to current life stressors and difficulties that are directly or indirectly related to psychiatric symptoms.^26^ The first few sessions of PCT provide psychoeducation about the typical symptoms and features associated with suicidal thoughts and behaviors. Within the current study, patients also completed a safety plan during the first session. Subsequent sessions are less structured and focus on topics chosen by the patient, who is encouraged to record in a journal any issues experienced between sessions. Therapeutic interventions include active listening, emotional validation, supportive counseling, and encouraging problem-solving to enhance coping, but no systematic training in behavioral or cognitive strategies for managing emotions or changing suicide-focused thoughts was provided. PCT was used as an active comparator because it has been shown to reduce psychiatric symptoms like depression, posttraumatic stress disorder,^33^ and suicidal ideation,^24,25,34^ but does not contain the cognitive or behavioral skills training components central to BCBT.

### Therapist Training, Supervision, and Treatment Fidelity

Therapists included predoctoral psychology interns, clinical social workers and professional counselors, postdoctoral psychologists, and licensed clinical psychologists. All therapists completed a 2-day training for each therapy (4 days total) and participated in weekly, hour-long consultation and supervision. All therapy sessions were audio- or video-recorded for fidelity review purposes. Two complete cases for each treatment group (all 12 sessions) were reviewed for fidelity unless the therapist saw less than 2 therapy participants within a condition. In addition, a small proportion of sessions (approximately 15%) were selected at random to monitor ongoing fidelity. Mean (SD) treatment fidelity scores across all clinicians were 92.7% (0.1%) for BCBT and 96.4% (0.1%) for PCT.

### Outcomes

#### Primary Outcome: Suicide Attempts

Suicide attempts were assessed using the self-report version of the SITBI-R,^28^ an evidence-based instrument that distinguishes among different types of suicidal and self-injurious behaviors. In this study, suicide attempts included aborted, interrupted, and actual suicide attempts.

#### Secondary Outcome: Suicidal Ideation

Severity of suicidal ideation was assessed using the self-report SSI,^27^ a psychometrically validated instrument that measures the intensity of suicide-related thoughts, urges, intentions, and behaviors. Items are summed to provide a continuous indicator of suicidal ideation, with higher scores indicating more severe suicidal ideation.

### Sample Size Estimation

For our primary end point, suicide attempts, we used SAS version 9.4 PROC POWER (SAS Institute) to estimate the minimum required sample size to detect a statistically significant hazard ratio (HR) of 0.5 (ie, 50% difference in suicide attempt rates) with 80% power, a 1-sided α = .05, and 20% attrition (see Supplement 1 for additional details of a priori power analysis). Results indicated a sample size of 82 (41 per group) yielded 80% power when the suicide attempt rates across groups were comparable to those reported in several previously published trials (ie, 20% vs 40%).^6,8,35,36,37^ For our secondary outcome, suicidal ideation, we used RMASS version 2 (Center for Health Statistics) to estimate the minimum required sample size to detect a small to medium between-group standardized mean difference (*d* = 0.3) in suicide ideation when using a mixed-effect model with 5 assessments (baseline, 3 months, 6 months, 9 months, and 12 months) and an expected autocorrelational structure with *r* = 0.2 between adjacent measurements. Results indicated a sample size of 84 yielded 80% power.

### Statistical Analysis

All analyses used the intent-to-treat principle, which included all participants randomized to each treatment group regardless of dropout, withdrawal, or protocol adherence.^38^ Baseline demographic and clinical variables were compared across treatment groups to identify potential covariates in the primary analyses. For hypothesis 1, we compared the percentages of participants making at least 1 follow-up suicide attempt and the total number of follow-up suicide attempts made in each treatment group. To compare the percentages of participants who attempted suicide, we used the Kaplan-Meier method^39^ and Cox regression models with time to suicide attempt as the outcome, where time was calculated as the number of days from the baseline screening appointment to each suicide attempt. The Kaplan-Meier and Cox regression model utilizes all available data from all participants regardless of dropout or length of follow-up; they are therefore robust to missingness and align with the intent-to-treat principle. Cox models also allow for the specification of covariates. To compare the total number of follow-up suicide attempts across groups, we used Poisson count regression, which assumes uniform risk of recurrent attempts over time, with treatment group as the exposure variable and the Andersen-Gill counting process model,^40^ an extension of Cox regression that assumes the risk of recurrent suicide attempts within a participant is related to an underlying vulnerability that persists over time, but each attempt does not depend on the occurrence of previous attempts.^41^ For hypothesis 2, we used generalized linear mixed-effects regression models with repeated measures using an unstructured covariance matrix, selected based on likelihood criteria (Bayes Information Criterion). SSI score was treated as a continuous outcome, while treatment group, time, and their interaction terms were entered as fixed-effect variables, and random effects were specified for the intercept and slope. Number of prebaseline suicide attempts and personality disorder diagnoses significantly differed between groups; these variables were therefore included as covariates in the Cox and mixed-effects regression models. All analyses were conducted using SAS version 9.4 with α = .05 a priori significance levels and 2-sided hypothesis tests. Analysis was conducted from April to September 2024.

## Results

**Figure 1.**
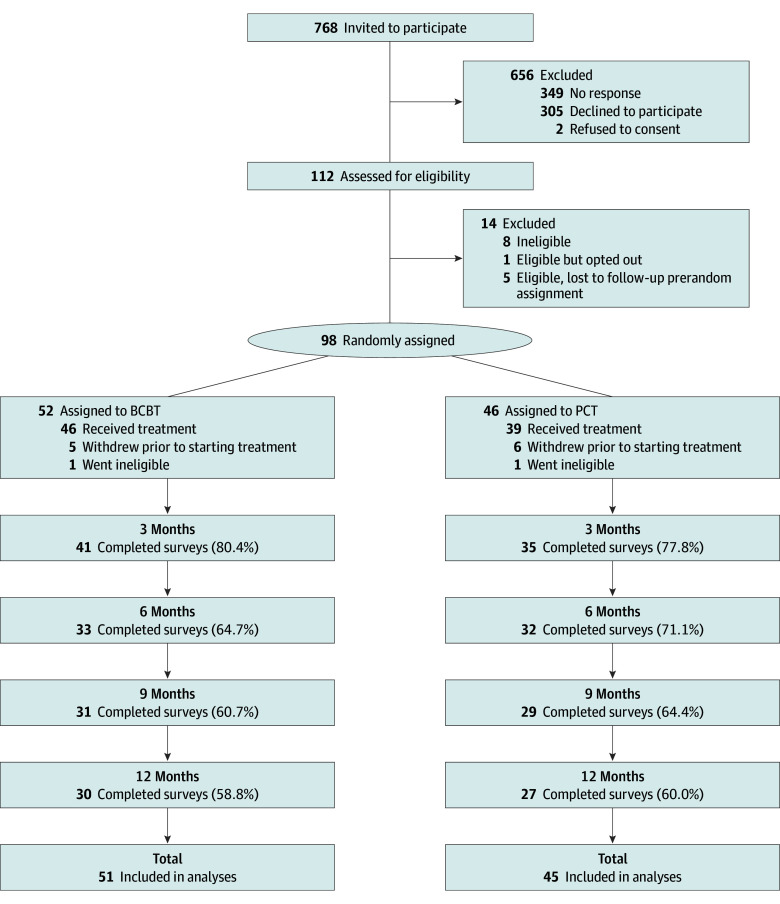
Participant Flow Through the Study BCBT indicates brief cognitive behavioral therapy; PCT, present-centered therapy.

A total of 96 adults seeking treatment (mean [SD] age, 31.8 [12.6] years; 64 female [66.7%] and 32 males [33.3%]) were included in the study, with 51 receiving BCBT and 45 receiving PCT (Figure 1 and Table 2). Ten participants (10.4%) had attempted suicide once prior to enrollment and 36 (37.5%) had previously attempted suicide 2 or more times. The mean (SD) SSI score at baseline was 15.0 (6.8). At baseline, the treatment groups did not differ on any demographic variable or severity of suicidal ideation. As compared with participants receiving BCBT, participants receiving PCT were more likely to have a personality disorder diagnosis (13 participants [33.3%] vs 4 participants [8.7%]; χ^2^_1_ = 7.7; *P* = .005) and reported a greater median (IQR) number of prior suicide attempts (6 [1-13] attempts vs 3 [0-7] attempts; Wald χ^2^_1_ = 8.1; *P* = .004).

**Table 2.** Participant Characteristics

Characteristic	Participants, No. (%) (N = 96)	
	BCBT (n = 51)	PCT (n = 45)
Sex		
Female	32 (62.7)	32 (71.1)
Male	19 (37.2)	13 (28.9)
Gender		
Woman	30 (58.8)	30 (66.7)
Man	18 (35.3)	13 (28.9)
Transgender	3 (5.9)	2 (4.4)
Sexual orientation		
Straight	27 (52.9)	25 (55.5)
Bisexual	10 (19.6)	9 (20.0)
Gay or lesbian	6 (11.7)	3 (6.7)
Something else	5 (9.8)	3 (6.7)
I am not sure yet	0	2 (4.4)
Refused	0	1 (2.2)
Missing	3 (5.8)	2 (4.4)
Race		
African American or Black	3 (5.8)	3 (6.7)
American Indian or Alaska Native	0	1 (2.2)
Asian	13 (25.5)	2 (4.4)
Pacific Islander or Native Hawaiian	1 (1.9)	0
White	31 (68.9)	36 (80.0)
Other^a^	3 (5.8)	3 (6.7)
Hispanic or Latino ethnicity		
No	50 (98.0)	44 (97.8)
Yes	0	1 (2.2)
Missing	1 (2.0)	0
Prior suicide attempts, No.		
0	25 (49.0)	25 (55.6)
1	7 (13.7)	3 (5.9)
≥2	19 (37.3)	17 (37.8)
*DSM-5* diagnosis		
Depressive disorders	43 (93.5)	28 (71.8)
Trauma- and stressor-related disorders	9 (19.6)	13 (33.3)
Substance use disorders	1 (2.2)	2 (5.1)
Anxiety disorders	26 (56.5)	20 (51.3)
Bipolar and related disorders	3 (6.5)	2 (5.1)
Obsessive-compulsive and related disorders	0	3 (7.7)
Personality disorders	4 (8.7)	13 (33.3)
Feeding and eating disorders	0	1 (2.6)
Neurodevelopmental disorders	3 (6.5)	1 (2.6)
Intermittent explosive disorder	1 (2.2)	2 (5.1)
Psychiatric hospitalizations during study	1 (1.9)	4 (8.9)
Age, mean (SD), y	33.0 (12.9)	30.7 (12.3)
No. of prior suicide attempts, median (IQR)	3 (0-7)	6 (1-13)

Abbreviations: BCBT brief cognitive behavioral therapy; *DSM-5*, *Diagnostic and Statistical Manual of Mental Disorders* (Fifth Edition); PCT, present-centered therapy.

^a^ Other was a formal category that included any race not otherwise specified.

### Attrition

A total of 768 participants were invited to participate, 112 were assessed for eligibility, and 98 participants were randomized to either BCBT (52 participants) or PCT (46 participants) (Figure 1). Dropout rates were similar across treatments, with 5 patients receiving BCBT (9.6%) and 6 patients receiving PCT (13.0%) who dropped out prior to starting treatment. Following randomization, 1 participant receiving BCBT (1.9%) was found to be ineligible for continuation in the study due to insufficient English proficiency and 1 participant receiving PCT (2.2%) became ineligible due to an inability to access telehealth sessions through a smartphone, tablet, or computer. A total of 85 participants (88.5%) completed at least 1 session. Eighty participants (83.3%) completed any follow-up assessment across all 4 time points for our primary end point. Fourteen participants receiving BCBT (29.8%) and 12 participants receiving PCT (30.8%) PCT attended fewer than 12 sessions. Of those who attended fewer than 12 sessions, most (15 participants [57.7%]) dropped out by the third therapy session.

### Primary Outcome: Suicide Attempts

**Figure 2.**
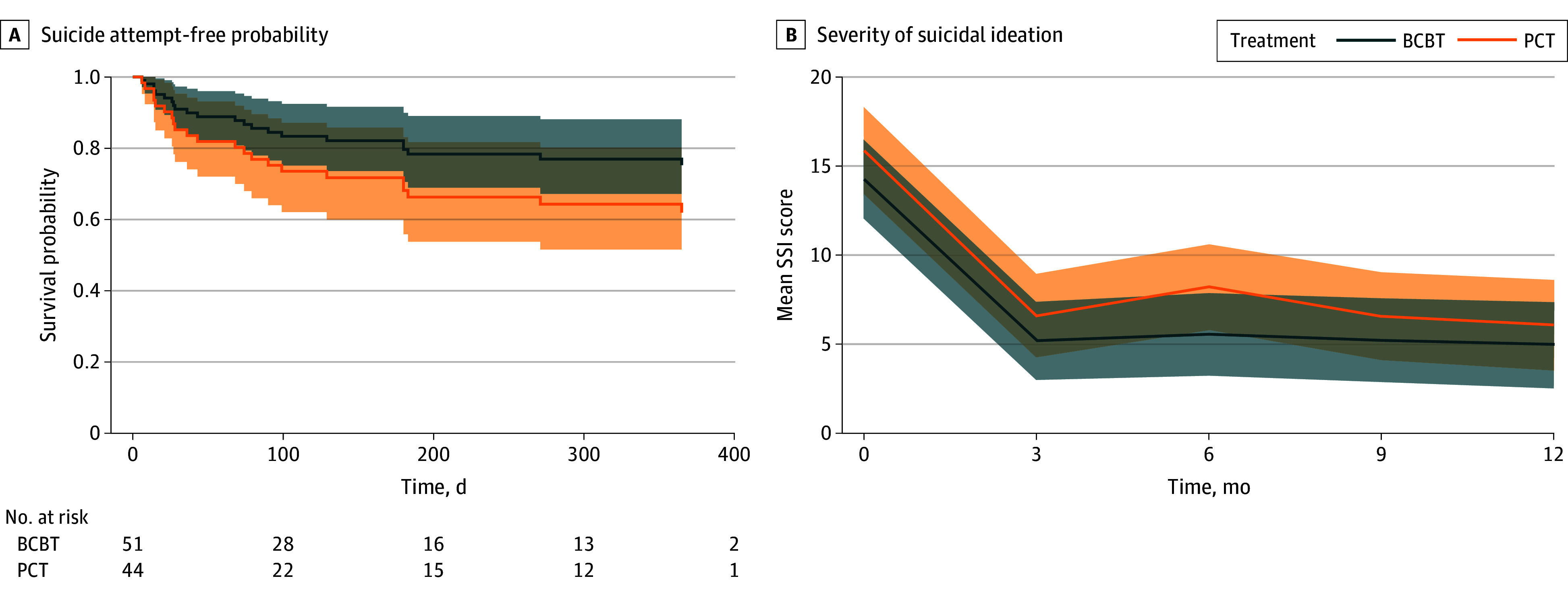
Suicide Attempt-Free Probability and Severity of Suicidal Ideation Over Time Among High-Risk Patients Receiving Brief Cognitive Behavioral Therapy (BCBT) vs Present-Centered Therapy (PCT) via Video Telehealth BL indicates baseline; SSI, Scale for Suicide Ideation.

Follow-up suicide attempts by group are summarized in Table 3. From baseline to the 12-month assessment, 12 participants receiving PCT (estimated percentage, 35.6%) made 56 suicide attempts (30 aborted, 19 interrupted, and 7 actual) and 11 participants receiving BCBT (estimated percentage, 30.0%) made 36 suicide attempts (25 aborted, 6 interrupted, and 5 actual). The percentage of participants in each group with a follow-up suicide attempt did not differ (log-rank χ^2^_1_ = 0.4; *P* = .50; HR, 0.91; 95% CI, 0.37-2.27; *P* = .84). Poisson model results indicated there were fewer suicide attempts in BCBT (mean [range], 0.70 [0.00-8.00] attempts per participant; 95% CI, 0.49-1.00 attempts per participant) than PCT (mean [range], 1.40 [0.00-10.00] attempts per participant; 95% CI, 1.07-1.84 attempts per participant) (Wald χ^2^_1_ = 9.3; *P* = .002) (Table 3). The Anderson-Gill model indicated participants randomized to BCBT had an approximately 41% reduced risk for suicide attempts compared with participants in PCT (HR, 0.59; 95% CI, 0.36-0.96; *P* = .03) (Figure 2A).

**Table 3.** Suicide Attempt Counts and Means Across Groups, by Attempt Type

Attempt type	Suicide attempts, Total No. (mean per participant) [95% CI]	
	BCBT	PCT
Combined	36 (0.70) [0.49-1.00]	56 (1.40) [1.07-1.84]
Aborted	25 (0.51) [0.33-0.78]	30 (0.73) [0.50-1.06]
Interrupted	6 (0.09) [0.04-0.24]	19 (0.47) [0.29-0.74]
Actual	5 (0.10) [0.04-0.24]	7 (0.19) [0.09-0.39]

Abbreviations: BCBT brief cognitive behavioral therapy; PCT, present-centered therapy.

### Secondary Outcome: Suicidal Ideation

As shown in Figure 2B, severity of suicidal ideation significantly decreased in both treatments (*F*_4,330_ = 50.1; *P* < .001). The slopes did not differ between groups (*F*_4,330_ = 0.2; *P* = .91).

## Discussion

In this RCT of adult psychiatric outpatients at elevated risk for suicide, BCBT delivered via telehealth significantly reduced suicide attempts by approximately 41% when compared with PCT. Significant reductions in suicidal ideation severity were observed in both groups and did not differ between BCBT and PCT.^8,35^ To our knowledge, this is the first study supporting the efficacy of any suicide-focused psychotherapy delivered via telehealth. Consistent with previous studies,^6,8,35,36^ significant reductions in suicide attempts occurred despite minimal between-group differences in severity of suicidal ideation, suggesting the mechanisms underlying reductions in suicidal behaviors are different from those underlying reductions in suicidal ideation.^42^ Although additional research is needed to distinguish the therapeutic targets associated with suicidal behaviors vs ideation, a key implication of this finding is that suicidal ideation may have limited clinical utility as an indicator of treatment response and risk for suicidal behavior. Current approaches for assessing suicidal ideation may also be limited and unable to fully capture the variable nature or critical elements of ideation.^43^

A strength of this study is the use of an active, evidence-based treatment as the comparator instead of treatment as usual.^6,7,8^ The use of an active comparator in this study provides a higher level of internal validity than previous studies, thereby enabling us to conclude with greater confidence that reductions in suicide attempts are likely attributable to the skills-training focus of BCBT, which prioritizes targeting core underlying vulnerabilities in how patients regulate emotions and cognitively reappraise stressful situations. BCBT teaches patients how to reduce autonomic arousal (eg, relaxation) and rumination (eg, mindfulness), increase positive affect (eg, reasons for living or activity planning), and change extreme negative beliefs about themselves and the world (eg, cognitive reappraisal). The structured approach of BCBT, compared with the less structured format of PCT, may also contribute to the effectiveness of BCBT in reducing suicide attempts. The effects of BCBT may also be attributable to crisis response planning, a central component of BCBT introduced in the treatment’s first session, which has been shown in multiple RCTs to significantly reduce suicide attempts.^37,44^ Additional research is needed to further isolate the effects of BCBT and identify its most active components.

### Limitations

Conclusions based on this study should be made with the following limitations. Of note, this study enrolled a relatively small number of patients who lived in predominantly urban and suburban areas with reliable access to broadband internet service. These results should therefore be considered preliminary until replicated in larger and more diverse samples, including people in rural areas or without high-speed internet access. Second, because we assessed follow-up suicide attempts via self-report only, we may have undercounted suicide attempts among participants who did not complete the follow-up assessments. It is also possible that there were treatment differences that made participants in one group compared with another more inclined to report suicide attempts. Another limitation is that this study was conducted primarily during the worldwide COVID-19 pandemic and outcomes could vary if replicated postpandemic.

## Conclusions

The present results provide further support for the effectiveness of BCBT for preventing suicide attempts among adults with elevated risk for suicide and indicate the treatment’s effect on reducing suicide attempts is preserved when delivered remotely via video-based telehealth. To our knowledge, this study is the first study to demonstrate that individuals at high risk of suicide can be safely and effectively treated via telehealth, which has implications for increasing access to care, while maintaining treatment fidelity with established evidence-based treatments. Additional research is needed to replicate and extend these findings and identify the mechanisms underlying the effects of BCBT.

## Notes

Supplement 1.
Trial Protocol

Supplement 2.
Data Sharing Statement
