# Supplementary material for: Telehealth Brief Cognitive Behavioral Therapy for Suicide Prevention: A Randomized Clinical Trial
Source: JAMA Netw Open. 2024 Nov 12;7(11):e2445913. doi: 10.1001/jamanetworkopen.2024.45913 (PMC11558477; doi:10.1001/jamanetworkopen.2024.45913)
Supplement: Supplement 1. — Trial Protocol [file jamanetwopen-e2445913-s001.pdf]

A randomized clinical trial testing the effectiveness of telemental health for suicidal patients

1. Objectives

The **long-term goal** of this study is to reduce suicidal thoughts and behaviors among treatment-seeking individuals who are experiencing suicidal thoughts or have recently made a suicide attempt. Brief cognitive behavioral therapies for suicide prevention (BCBT) has demonstrated empirical support for reducing suicide attempts as compared to treatment as usual (Brown et al., 2005; Rudd et al., 2015; Sinyor et al., 2020). However, no studies to date have assessed their effectiveness when delivered via telehealth, highlighting an important knowledge gap in light of increased use of telehealth subsequent to the outbreak of the novel coronavirus (COVID-19) in the U.S. In light of this knowledge gap, the **primary objective** of this study will be to test the effectiveness of brief cognitive behavioral therapy (BCBT) as compared to present-centered therapy (PCT), an active comparator, for the reduction of suicide ideations and attempts when delivered via telehealth. To accomplish these objectives, we will enroll individual participants who have reported recent suicidal ideation and/or suicidal behaviors. We will utilize self-report and interview methods to compare treatment effects. To achieve our primary objective, we specifically propose to:

Aim 1: Investigate the efficacy of BCBT-T for the prevention of suicide attempts in a psychiatric outpatient sample.

H1: Individuals receiving BCBT condition will have significantly reduced suicide ideation and suicide attempt during follow-up as compared to individuals receiving PCT.

Aim 2: Identify cognitive-affective mediators of BCBT's effects on risk for suicide attempt, when delivered via telehealth.

H2a: Individuals receiving BCBT condition will show significantly larger improvements in emotion regulation and decision-making style than individuals receiving PCT.

H2b: The effect of BCBT on reduced suicide ideation and attempts will be mediated by improvements in emotion regulation and decision-making style.

H2c: Emotion Artificial Intelligence (AI) will identify recognizable emotional patterns associated with various stages of both psychotherapies as it pertains to participant outcome variables.

2. Background and Rationale

Suicide remains one of the top 10 causes of death in the United States. Since 1999, the age-adjusted suicide rate has increased by approximately 33%, from 10.5 per 100,000 in 1999 up to 14.0 per 100,000 in 2017 (Hedegaard, Curtin, & Warner, 2018). Cognitive behavioral therapies are empirically supported for the rapid reduction of suicidal thoughts and behaviors, especially when they are delivered in an individual therapy (versus group therapy) format (Tarrier et al., 2008). One particular protocol—brief cognitive behavioral therapy for suicide prevention (BCBT)—was developed by our research team and tested in a sample of active duty military personnel in a randomized clinical trial. Results of that study showed that service members receiving BCBT were 60% less likely to attempt suicide

during the two-year follow-up and showed faster reductions in suicide ideation as compared to service members receiving treatment as usual (TAU; Rudd et al., 2015). A recently completed pilot randomized clinical trial similarly found a large reduction in suicide attempt rates among adolescents and young adults who received BCBT as compared to TAU (0% vs. 25%; Sinyor et al., 2020).

Unfortunately, fewer than half of suicide decedents and individuals with past-year suicide ideation seek out mental health treatment (Hom, Stanley, & Joiner, 2014). While several factors influence the decision to use (or not use) mental health services, logistical barriers such as insufficient time and/or geographic location are among the most commonly cited reasons for choosing not to access treatment. Telehealth services can address this disparity due to reduced need for travel and less time away from school and/or work, which can increase attendance rates to medical appointments (Saeed, Diamond, & Bloch, 2011). Evidence also suggests that delivering mental health services via telehealth improves help-seeking behaviors (Kauer, Managan, Sanci, 2014). Although numerous studies indicate that the efficacy of empirically-supported treatments for a wide range of mental health conditions are not diminished when delivered via telehealth (e.g., Osenbach et al., 2013; Sloan et al., 2011), no studies have examined the effects of suicide-focused treatment protocols when delivered in this format.

The present study aims to address this knowledge gap by testing the effectiveness of BCBT when delivered via telehealth as compared to present-centered therapy (PCT), an active comparator that has been shown to significantly reduce suicide ideation (Bryan et al. 2016; Resick et al., 2017). The results of this study would provide critical information about the effectiveness of BCBT when delivered via telehealth.

Emotion Analytics in Support of Mental Health Outcomes

With the explosion of artificial intelligence (AI) in recent years and the continued shortage of providers to fill the mental health treatment gap, healthcare has seen a growing investment in emotion analytics to improve clinical outcomes. The COVID-19 pandemic further exposed the need for remote patient monitoring, point-of-care diagnosis, and virtual care. Evidence has come to support the use of machine learning and emotion artificial intelligence (Emotion AI) to measure, detect, and interpret emotional and behavioral changes in psychotherapy (Gual-Montolio et al, 2022; Miner et al, 2022; Tanana et al, 2021; Taubitz et al, 2022); to streamline diagnosis and clinical assessment (De Choudhury et al, 2021; Guntuku et al, 2017; Zhang et al, 2022); and to improve risk prediction models for suicide prevention (Kusuma et al, 2022; Walsh et al, 2017). Extant literature offers diverse applications of innovative methods to support long-standing needs, and the merging of clinical and automated assessments are among these innovations (Henry et al, 2022).

Clinical assessments and automated methods have both been found to be effective approaches in detecting suicidal ideation. Clinical assessments often rely upon self-report surveys and measurements to identify mental illness and related symptomatology, for example, risk of harm to self or others. This approach is based upon several assumptions, including that the person is aware of unfolding thoughts and feelings; that the person will be able to verbally communicate the experience in varied emotional states; and that the person will be motivated to answer honestly (Barron, 2021). In contrast, automated methods have become particularly adept at identifying and diagnosing mental illnesses through the study of naturally unfolding expressions in textual communications (Chancellor & Choudhury,

2020). Online usage such as social media posts and Google search terms have demonstrated the ability to better predict completed suicides than conventional self-report measures (Ma-Kellams et all, 2015; Ophir et al, 2020), and machine learning models have been found to improve traditional suicide prediction models (Kusuma, 2022).

Diverse treatment modalities including cognitive-behavioral therapy, emotion-focused therapy, and psychodynamic therapy have been shown to facilitate therapeutic change through a reformulation of past emotional memories and transformation of present emotional experience (Greenberg et al, 2007; Lane et al, 2015). Given that emotions play a central role in psychotherapy outcomes, significant consideration must be made to systematically explore patterns of emotional engagement, emotional expression, and emotion regulation in psychotherapy sessions (Sloan and Kring, 2007). Frequently, the study of emotion in psychotherapy has been bound by methodological limitations, as analyses have typically involved human raters and been largely retrospective, time-consuming, and subjective (Tanana et al, 2021). Recent developments in machine learning and natural language processing (NLP) provide an avenue for large-scale, systematic, real-time measurement of emotion in narrative. Cognovi Labs' Emotion AI is a contemporary application of NLP and behavioral psychology.

Emotion Artificial Intelligence

Cognovi Emotion AI™ is a technology that incorporates a behavioral psychology layer in its machine learning algorithms (Cognovi Labs, 2023). By applying Emotion AI to any textual or transcribed data, a user can obtain the underlying emotions of a given communication. Discrete emotion categories measured by Cognovi Emotion AI™ include joy, anger, disgust, fear, sadness, surprise, amusement, contempt, hope, and trust. While the first six are considered *basic* or *primary emotions* originally set forth by Ekman and Friesen (1971), the latter are *complex* or *secondary emotions* which generally evolve in an interpersonal context and can be heavily impacted by experience, expectations, and biases (Becker-Asano & Wachsmuth, 2008). Contemporary emotion theory underscores the functions served by emotions (Barrett & Salovey, 2002; Frijda, 1994).

By tuning into an individual and a therapeutic dyad's emotional expressions, Emotion AI detects nuanced fluctuations in emotion over time and highlights the arising activation. It identifies critical moments that require further attention, as emotions and action tendencies are intimately intertwined (Lowe & Ziemke, 2011), and combinations of emotions are related to impending actions (Fontaine & Scherer, 2013; Matsumoto et al., 2015). Action tendencies are states of readiness to execute a given action, for example moving closer to an object, connecting with it, interfering with it, or simply observing. Cognovi Labs has mapped 10 emotions onto 10 action tendencies, and organized the action tendencies along broader categories, or Action Personas, that capture their position on a continuum: Withdraw, Consider, Approach, and Tackle.

Withdraw: Feeling helpless, powerless, or ineffective, so staying passive, holding back, or pulling away. The person wants nothing to do with someone or something, so withdraws from the situation.

Consider: To protect oneself or others, a person pauses to observe or further witness a situation. The person actively contains internal reactions and considers whether or not to engage.

Approach: The act of seeking to approach, connect, and stay close to a person, place, or thing. The person feels ready to invest time and resources to understand the options and

pursue action.
Tackle: Feeling stimulated, motivated, and ready to oppose an obstacle or conquer a challenge. The person is inclined to antagonize, provoke, or tackle one's goal.

Downstream from classifying Action Personas, Cognovi Labs also utilizes two summary metrics designed to allow for rapid interpretation of isolated emotions: *Emotionality* and *Intent*. Emotionality represents the proportion of content that contains detected emotions. Intent describes the level of activation, or the inherent likelihood of action as a consequence of an underlying emotional state. Secondary outcomes, in part, will focus on evaluating the ability of Cognovi Emotion AI to identify recognizable emotional patterns associated with various stages of both BCBT and PCT as it pertains to participant outcomes (i.e., discontinuing sessions, progress in therapy, continued suicidal ideation, etc.).

3. Procedures

3.1. Research Design

The study includes a two-arm randomized clinical trial of BCBT as compared to PCT. The primary outcomes will be suicide ideation and attempts during a one-year follow-up period. Outcomes will be assessed using a combination of interview,self-report data, and natural language processing of session transcripts.

3.2 Sample

We plan to enroll 160 adults reporting suicidal ideation with intent to die within the past week or a suicide attempt within the past month.

3.3 Recruitment

3.3.1. Potential participants will be identified by clinical staff affiliated with the OSU Harding Hospital and Talbot Hall mental and behavioral health services. Patients with active suicide ideation or a recent suicide attempt will be referred by clinicians to a member of the study team to determine their eligibility. Potential participants will be identified through self-report of suicidal thoughts or behaviors on existing suicide risk screening and assessment tools currently in use across OSUWMC's mental and behavioral health services.

3.3.2.. We will post advertisements on social media sites (e.g., Facebook, Twitter), online advertising boards (e.g., Craigslist), in digital and print media (e.g., newspapers, newsletters, distribution of physical study flyer) within the community.

3.4 Eligibility Criteria

Our **inclusion criteria** will include the following: (1) 18 years of age or older; (2) a score of 5 or higher on the Scale for Suicide Ideation and/or a suicide attempt within the past month; (3) ability to understand and speak the English language; (4) ability to complete the informed consent process; (5) regular access to a stable internet connection; and (6) ownership of an internet-enable communication device (e.g., computer, tablet, smartphone). Our **exclusion criteria** will include the following: (1) substance use disorder requiring acute medical management; (2) imminent suicide risk warranting inpatient hospitalization; and (3) impaired mental status that precludes the ability to provide informed consent (e.g., intoxication, psychosis, mania).

3.5 Treatment Conditions

In the proposed study, participants will be randomized to receive one of two treatments to be delivered via telehealth: brief cognitive behavioral therapy (BCBT) or present-centered therapy (PCT). Both treatment conditions will include 12 outpatient individual psychotherapy sessions scheduled on a weekly or biweekly basis, with the first session lasting 90 minutes and subsequent sessions lasting approximately 60 minutes. Specific procedures and components contained within each treatment are summarized below in Table 1.

3.5.1. BCBT is divided into three phrases. In phase I (5 sessions), the therapist conducts a detailed assessment of the patient's most recent suicidal episode or suicide attempt, identifies patient-specific factors that contribute to and maintain suicidal behaviors, provides a cognitive-behavioral conceptualization, collaboratively develops a crisis response plan, and teaches basic emotion regulation skills such as relaxation and mindfulness. In phase II (5 sessions), the therapist applies cognitive strategies to reduce beliefs and assumptions that serve as vulnerabilities to suicidal behavior (e.g., hopelessness, perceived burdensomeness, guilt and shame). In phase III (2 sessions), a relapse prevention task is conducted, during which patients imagined the circumstances of a previous suicidal episodes and the internal experiences associated with this event (i.e., thoughts, emotions, physiological responses), and then imagine themselves using one or more skills learned in BCBT. Participants then imagine themselves successfully resolving hypothetical future crises. Participants must demonstrate the ability to successfully complete this task in order to terminate the treatment. Additional sessions are conducted until participants demonstrate the ability to complete this task.

3.5.2. PCT includes (1) psychoeducation about the typical symptoms and features associated with suicidal thoughts and behaviors; (2) normalization of symptoms; (3) experience of receipt of support and feedback from a licensed professional; and (4) positive interpersonal interactions. PCT differs from BCBT in several key ways including less structure (i.e., patients are allowed to have more input into PCT session agendas) and no systematic training in behavioral or cognitive strategies for managing emotions and changing suicide-focused thoughts. PCT will be used as an active comparator because it is an empirically supported treatment for depression and PTSD that also reduces suicidal ideation (Bryan et al., 2016; Resick et al., 2017), but contains unique elements that distinguish the treatment from BCBT (see Table 1).

Table 1. Treatment procedures contained within BCBT and PCT

Treatment Procedure/Component	BCBT	PCT
Suicide risk screening	X	X
Narrative assessment	X	
Crisis response plan / safety plan	X	X
Means safety counseling	X	X
Weekly monitoring of suicide risk	X	X
Psychiatric symptom management	X	X
Psychoeducation: suicide as symptom of mental illness		X
Psychoeducation: suicide as a deficit in self-regulation	X	
Emotion regulation skills training	X	
Cognitive restructuring skills training	X	

Relapse prevention task	X	

To control for therapist confounding, both BCBT and PCT will be administered by the same research therapists. All BCBT and PCT sessions will be recorded via the Zoom platform to ensure fidelity and to prevent cross-contamination of therapies. Recordings will be reviewed and scored using fidelity monitoring checklists specific to each therapy by the investigators. Research therapists will participated in weekly supervision to receive feedback.

To minimize bias associated with expectancy effects, participants will not be informed about their treatment group assignment. Treatments will instead be referred to as Treatment A and Treatment B.

3.6 Clinician Training and Supervision

Research therapists will complete two-day trainings in both therapies (BCBT and PCT) with follow-up consultation and supervision. All therapy sessions will be audio or video recorded, and a random sample will be selected for fidelity monitoring and review by members of the investigative team who are approved trainers and consultants for each procedure. Following each therapy session, clinicians will upload their electronic recording to a shared, access-restricted folder on a secured OSU server. File names will comprise the participant's ID number, therapist's initials, and session number (e.g., 3298JB2).

3.7 Cognovi Emotion AI procedures

The research study's psychotherapy session transcripts will undergo emotional analysis in Cognovi Labs' Emotion AI technology. Drs. Nirit Pisano and Ryan Mann will facilitate the transfer of anonymized psychotherapy session transcripts from Ohio State University to Cognovi Labs. No personal identifiable information (PII) will be included in the transfer to maintain the privacy of study participants. As transcripts are received, they will be uploaded into Cognovi's Explorer technology, which handles large scale data sets and allows for both aggregation and parsing of transcripts. Within the Explorer, communications can be organized and emotions measured along diverse variables, for example by therapist, patient, session number, treatment modality, intervention type, diagnosis, topic of discussion (e.g. suicidality), gender, treatment length, and any other feature of interest. As the data is loaded, automated emotion tagging will assign each sentence with one or more of Cognovi Labs' 10 emotions. Emotion tagging occurs upon entry in real time. Topic clustering will identify emotionally salient issues in a single session or across entire treatments.

Transcripts will also run through Cognovi's Communicator technology, which allows for more in-depth analysis of a two-person interaction. The therapist-patient dyad can be evaluated as a single unit in order to determine the "emotional journey" of a session, an exchange, or an entire treatment. This will highlight any noteworthy rise or fall in emotional or behavioral metrics. Each speaker's emotional communication can also be analyzed in terms of what it evokes in the other, revealing a back-and-forth dynamic whereby certain emotions may be mirrored back while others are not.

3.7 Randomization

Individuals who meet inclusion criteria will be randomized to either BCBT or PCT using a stratified block randomization algorithm designed to balance participants across both conditions based on the following variables of interest: biological sex (male or female) and

history of suicide attempt (no previous attempts, one previous attempt, two or more previous attempts). To prevent the possibility of staff members manipulating participant assignment to treatment, a computerized randomization technique will be used.

3.8. Blinded Assessments

Follow-up assessments will be conducted by an independent evaluator who is blind to treatment assignment.

3.8 Sample Size Estimation

Previous studies indicate that suicide attempt rates during the first year postbaseline range from 7-25% among patients receiving CBT and from 20-50% among patients receiving treatment as usual. The lower bounds of these estimates occurred in a study of active duty military personnel with a predominantly male sample (Rudd et al., 2015). Follow-up analyses from that study (Bryan & Rudd, 2018) found higher suicide attempt rates among female participants in both treatment groups, comparable to the rates reported in other clinical trials that had majority (or entirely) female participants (Brown et al., 2005; Linehan et al., 2016, 2018). The sample to be enrolled in this study is expected to have a larger proportion of women than our earlier military study. Across all of these studies, CBT was associated with a 50% or larger reduction in suicide attempt rates as compared to treatment as usual. BCBT is also associated with significantly faster reductions in suicide ideation, with statistically significant and moderate between-group differences typically occurring 3-6 months postbaseline.

We used SAS 9.4 to estimate the minimum required sample size to detect a statistically significant log-rank test with 80% power and a two-sided alpha=.05 under a range of possible conditions (see Table 2). A total sample size of 160 (80 per arm) yielded sufficient power to detect a 50% reduction in suicide attempts when the observed rates were comparable to those reported in several previously published trials (20% vs. 40%).

Table 2. Results of power analyses for a range of suicide attempt rates across BCBT and PCT

BCBT	PCT	N
10%	15%	444
10%	20%	140
10%	30%	50
10%	40%	30
15%	20%	696
15%	30%	102
15%	40%	42
20%	30%	256
20%	40%	80

We used RMASS 2 to estimate the minimum required sample size to detect a statistically significant between-group difference in suicide ideation when using a mixed effect model with 5 assessments (baseline, 3 months, 6 months, 9 months, 12 months) and an expected autocorrelational structure with r=0.2 between adjacent measurements. Under these assumptions, a minimum of 76 participants (38 per arm) is required to detect a small to medium standardized mean difference (d=0.3) between groups. Assuming 20% attrition during follow-up, the minimum sample size needed to detect this effect size increases only slightly to 84 participants.

4. Measurement / Instrumentation

The planned assessment schedule for this study is displayed in Table 3.

Table 3. Planned Assessment Schedule

Measure	BL	Tx	3 Mo	6 Mo	9 Mo	12 Mo
SITBI-R	■		■	■	■	■
SSI	■	■	■	■	■	■
PROMIS-43	■	■	■	■	■	■
SCS-R	■	■	■	■	■	■
CEQ	■	■				
MCQ	■		■	■	■	■
DERS-SF	■		■	■	■	■
YSS			■	■	■	■
PTHI			■	■	■	■
DIAMOND	■					

4.1. Primary Outcomes: Suicide Ideation & Attempts

Suicide attempts will be assessed using the Self-Injurious Thoughts and Behaviors Interview-Revised (SITBI-R). The SITBI-R is an empirically-supported researcher-administered interview for assessing the characteristics and features of self-injurious thoughts and behaviors, and to distinguish different types of suicidal and self-injurious behaviors.

Severity of suicide ideation will be assessed using the Scale for Suicide Ideation (SSI). The SSI is an empirically-supported researcher-administered interview that assesses the intensity of suicide-related thoughts, urges, intentions, and behaviors. All participants complete the first 5 items. If a subject positively endorses either item 4 (active ideation) or item 5 (passive ideation), they are directed to complete an additional 14 items.

4.2. Secondary Outcomes

Psychiatric symptom severity will be measured using the Patient-Reported Outcomes Measurement Information System (PROMIS). PROMIS is a 43-item self-report measure that asks participants' health status in the following domains: physical function, anxiety, depression, fatigue, sleep disturbance, pain interference, and participation in social roles. Respondents are directed to rate the frequency of each symptom within the past 7 days on a 5-point scale ranging from 0 (never) to 4 (always).

Suicidogenic beliefs will be assessed with the Suicide Cognitions Scale-Revised (SCS-R). The SCS-R is an empirically-supported self-report scale that assesses the strength and severity of maladaptive beliefs that contribute to suicidal thoughts and behaviors (e.g., hopelessness, self-hatred, distress intolerance). Respondents are directed to rate the extent to which the agree or disagree with a series of 16 statements on a 5-point scale ranging from 0 (strongly disagree) to 4 (strongly agree).

Decision-making style will be assessed with the Monetary Choice Questionnaire (MCQ), a 27-item self-administered questionnaire in which the respondent chooses between a smaller, immediate monetary award and a larger, delayed monetary reward (e.g., "Would you prefer $14 today or $25 in 19 days?"). The method is scored by calculating where the respondent's answers place him or her relative to reference discounting curves, where placement among steeper curves indicates higher levels of impulsive decision-making

Emotion regulation will be assessed with the Difficulties in Emotion Regulation Scale Short Form (DERS-SF), an 18-item self-report measure that assesses six components of emotion

regulation deficits: nonacceptance of emotional responses, difficulties engaging in goal-directed actions, impulse-control difficulties, lack of emotional awareness, limited access to emotion regulation strategies, and lack of emotional clarity.

Treatment acceptability and impact will be assessed using items from the Youth Services Survey (YSS), a self-report scale that measures a patient's perceptions about mental health services and treatment, and perceived benefits resulting from the treatment.

Credibility and expectations about treatment will be assessed using the Credibility Expectancy Questionnaire (CEQ), a self-report measure typically administered at the beginning and end of treatment to assess a patient's perceptions about the legitimacy of each treatment.

Post-treatment follow-on services will be measured at the follow-up assessments by the Post-Treatment Health Interview (PTHI). This measure is utilized to assess participants' engagement in mental health treatment (e.g. inpatient, group, outpatient, medications, etc).

Cognovi Emotion AI. Situated at the intersection of behavioral psychology and deep machine learning, Cognovi Emotion AI™ measures and interprets emotional communication as it is expressed through written or spoken word. The technology was developed over eight years at Wright State University's KNO.E.SIS Center with funding from the National Science Foundation, Department of Energy, and US Air Force. Cognovi Labs' emotion algorithms have been pre-trained on datasets containing tens of millions of examples. In contrast to tonation or sentiment analysis, Cognovi Labs' Emotion AI was trained to measure emotion in context. It is therefore able to capture sarcasm, metaphor, humor, speech irregularities, and other linguistic elements. The technology was trained natively in 20 languages, avoiding translation and safeguarding the cultural and linguistic differences inherent in diverse languages. The accuracy of language-specific emotion models are verified with respect to human annotated benchmarks, based on a majority rule of native speakers.

4.3. Covariates

Psychiatric diagnosis will be assessed with the Diagnostic Interview for Anxiety, Mood, and OCD and Related Neuropsychiatric Disorders (DIAMOND), an empirically-supported structured diagnostic interview that assesses the most common mood, anxiety, and trauma-related psychiatric disorders to include major depression and suicidality.

5. Detailed Study Procedures

Patients accessing treatment from an OSUWMC mental or behavioral health service will be referred to our research staff for eligibility determination if they screen positive for suicidal ideation or verbally report recent suicidal thoughts and behaviors. Research staff will attend regularly scheduled clinical team meetings and discharge planning meetings to identify potential participants. Referred patients will be contacted by a member of the research team to explain the purpose of the study, study procedures, and possible risks and benefits. All patients will be contacted using the established study contact script. Interested patients will then complete an initial screening to determine if they meet eligibility criteria.

Patients who are eligible for the study will be informed of their eligibility and complete the informed consent process. Those who consent to participate will then complete a baseline

assessment involving self-report measures and clinician-administered interviews. After completing the baseline assessment, participants will be randomized to either BCBT or PCT using a computerized randomization procedure that stratifies participants by biological sex and history of suicide attempts. Participants will then be scheduled for their first treatment session.

Therapy sessions will be scheduled once per week. During the active treatment phase, participants will complete self-report assessments of suicide ideation and symptom severity prior to the start of each session. These data will be collected and stored in Redcap, a secure web-based data collection system designed for clinical trials.

Participants will also complete self-report assessments and clinician-administered interviews 3 months, 6 months, 9 months, and 12 months postbaseline. All self-report assessments used at follow-up will be collected and stored via Redcap, a secure web-based data collection system designed for clinical trials. Participants will receive a $50 Amazon.com gift card for each follow-up assessment they complete. With four follow-up assessments, participants could therefore receive up to $200 total in Amazon.com gift cards.

5.1 Potential Risks

5.1.1 Emotional discomfort during self-report measures and interviews. Participants could develop mild to moderate emotional discomfort or frustration associated with filling out questionnaires and/or answering interview questions that ask about suicide, psychological symptoms, and other stressful experiences. This potential risk is expected to be comparable to the discomfort experienced when talking with a friend or acquaintance about these same topics. If discomfort is experienced, it is not expected to be severe or to last for more than a few minutes.

5.1.2 Emotional discomfort during treatment. Participating in mental health treatment and research might increase some symptoms and increase the risk of feeling emotionally uncomfortable in the short term, which might increase the desire for suicide temporarily. This increase is usually not severe, however, and does not last long. Work with these interventions in previous studies, suggest participants typically experience *decreased* emotional distress immediately after receiving treatment. It is also possible that participants might be uncomfortable not knowing the name of the intervention they have been randomized to, but previous experience studying these interventions suggest that most participants are generally okay with this lack of knowledge.

5.1.3 Breach of confidentiality. Participants' confidentiality could be breached if their identities are inadvertently released or accessed by a third party. Participants could also be identified based on the content of their responses. This risk is expected to be low because the data are not stored or analyzed in ways that are likely to reveal a subject's identity. Breach of confidentiality could also occur if an external party of individual hacks into Zoom interface during a participant's therapy sessions.

5.2 Protections Against Risk

To minimize the risk of emotional discomfort associated with survey questions and interviews, we will fully describe to participants the nature of study procedures and the potential for distress will be fully described to subjects before they complete any procedures.

Moreover, participants will be reminded that they can choose to discontinue any assessment at any time if they become severely distressed or stop the interview and take a break. Research staff are instructed to closely monitor participants while they are completing assessments.

Because we will be recruiting subjects with a history of suicidal thoughts and behaviors, we expect some subjects to be experiencing elevated emotional distress throughout the duration of the study. To minimize risk associated with this issue, all participants will receive either a safety plan or crisis response plan during their first session, consistent with standard of care recommendations for the management of suicide risk. The investigators will also be available to meet with distressed participants for additional crisis interventions, where needed. All follow-up assessments will be conducted by clinical research staff with an advanced mental health degree (e.g., social work, clinical psychology) and previous experience working with acutely suicidal patients, to include conducting suicide risk assessments and crisis interventions over the phone and via telehealth platforms.

The PI has independently conducted multiple studies with acutely suicidal individuals, and has considerable experience managing suicide risk. If a participant reports suicide ideation, or a research staff member becomes aware that the participant is at imminent risk to harm himself/herself, the following questions will be asked to clarify the nature of risk (and to identify those at imminent risk requiring consideration for hospitalization):

(1) Do you have a plan for killing yourself and do you intend to act on the plan?
(2) Do you have a desire to kill yourself that you believe you might act on?
(3) Have you already taken steps to act on your plan? If so, what steps have you taken?

Positive endorsement of any of the above items will be considered for potential hospitalization with additional assessment to be conducted by the research therapist. The research therapist will weigh all clinical indicators of risk and will make a clinical determination for hospitalization if warranted.

It is also possible for subjects to be identified as imminent risk if they indicate a moderate to severe level of suicide intent on the Beck Scale for Suicide Ideation (a score of 2 on Item 4), requiring evaluation for possible hospitalization. Once a participant is identified as potentially imminent risk, the researcher will conduct a more thorough assessment for possible hospitalization. If hospitalization is not indicated, the research clinician will review with the participant the steps contained within their crisis response plan / safety plan, a risk management procedure contained within each treatment group.

To minimize the risk of confidentiality breach, research staff will receive rigorous training in confidentiality and privacy procedures. Subject identifiers (e.g., name, date of birth, etc.) will also be stored separate from their data.
Coded audio/video recordings, transcripts, and other metadata associated with the recordings which will be utilized for the purposes of data analyses and fidelity monitoring will be stored by subject ID on HIPAA compliant servers.

Identifiable data will destroyed upon the completion of the study. We will use recommended security procedures and strategies for maximizing confidentiality and minimizing the risk of third-party intrusion during Zoom-based research activities. Subject data will be tracked using a sequential numeric ID system generated for each subject (e.g., 1001, 1002, 1003…). Finally, data will be stored in a deidentified manner using subject IDs instead of potential identifiers. De-identified transcripts (i.e., transcripts with identifying data such as name, date of birth, etc. removed using the Safe Harbor method) will additionally be stored by subject ID number. Only de-identified data may be shared with researchers on an individual basis. At the conclusion of the study, all identifiable data to include video recordings will be destroyed. De-identified transcripts will be maintained after the completion of the study. De-identified transcripts may be shared with external collaborators via approved methods after agreements created by the OSU Technology Commercialization Office (TCO) are finalized.

6. Data Analysis

Prior to statistical analyses, the data will be screened to assess the need for scale transformations (e.g., log, reciprocal) to normalize distributions or reduce variance heterogeneity. The study design is a two-arm randomized clinical trial with a superiority hypothesis (i.e., BCBT superior to PCT). Analyses will use survival analysis and mixed effects regression with repeated measures. Most participants will have 5 post-intervention assessments (baseline, 3 months, 6 months, 9 months, 12 months). Follow-up assessment data will be collected from participants, via electronically delivered links to the participants email via Redcap, even if they drop out of therapy early. The primary focus will be on overall group differences over time. Separate univariate analyses will be conducted with each primary outcome variable at unadjusted, two-tailed alpha=.05. Little's MCAR test will be used to determine if data are missing at random, and random effects pattern-mixture modeling will be used to determine if missingness influences longitudinal results. If data are not missing at random, we will use full information maximum likelihood estimation. The principal statistical software to be used is SAS 9.4 supplemented with other statistical software such as SPSS.

6.1 Hypothesis 1: Individuals receiving BCBT condition will have significantly reduced suicide ideation and suicide attempt during follow-up as compared to individuals receiving PCT.

We will analyze follow-up suicide attempt data using survival analysis with time-to-event data. The overall test of differences between the two intervention groups will be accomplished using the hazard ratio derived from the Cox regression and the log-rank and Wilcoxon statistics derived using the Kaplan-Meier method. Proportional hazard survival regression will also be used to explore between-group differences using demographic and baseline characteristics as covariates.

6.2 Hypothesis 2a: Individuals receiving BCBT condition will show significantly larger improvements in emotion regulation and decision-making style than individuals receiving PCT.

To test differences in continuous outcomes between groups over time, mixed effects regression models with repeated measures will be used. Treatment group will be the primary independent variable, and outcome measures at baseline and each follow-up assessment period will be entered as dependent variables. Covariance structures will be selected by comparison of likelihood criteria (e.g., Akaike's Information Criteria), though the autoregressive structure is well-suited for such analyses.

6.3 Hypothesis 2b: The effect of BCBT on reduced suicide attempts will be mediated by improvements in emotion regulation and decision-making style.

Figure 1.
The statistical mediation model

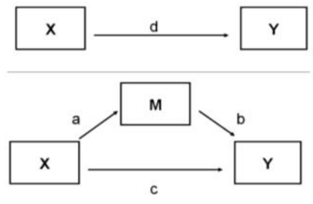

Figure 1 depicts statistical mediation as originally described by Baron & Kenny. The top panel shows a direct effect of variable X on Y, quantified by a single regression coefficient (d). The bottom panel depicts statistical mediation, the indirect effect of X on Y. In this model, the mediator (M) is a cause of Y, and a change in X causes change in the mediator. In this project, the causal variable (X) is intervention group. The mediator variables (M) are emotion regulation (measured with the DERS-SF), and decision-making style (measured with the MCQ). The mediation path is the product of coefficients a and b in the lower panel, so a statistical test of this multiplicative path a*b is a test of the significance of mediation. We will use regression parameters and their standard errors with 10,000 bootstrapped resamples as recommended by Preacher and Hayes to accomplish this analysis.

6.4 Hypothesis 2c: Data analysis will reveal the volume of overall communication, degree of emotional expression (Emotionality), extent of activation (Intent), and level of Action Personas (Withdraw, Consider, Approach, or Tackle). For example, high levels of anger, contempt, and amusement often lead to a more confrontational stance and therefore higher tackle, while significant joy, hope, and trust reflect an inclination to embrace, suggesting a readiness to approach. Emotional shifts throughout the transcript will lead to fluctuating metrics that will be assessed in regard to predefined variables.

We intend to utilize a nonexperimental quantitative design to investigate the underlying emotional patterns associated with each subject. Emotional patterns are to be established by our algorithm, Cognovi's Emotion AI™. Each emotional pattern will be calculated at both individual and group levels. Cognovi's Emotion AI™ provides quantitative metrics as previously set forth. These metrics will serve as baseline indicators for emotional patterns. The quantitative outputs will be statistically analyzed for outliers, trends, and anomalies using *R* statistical software (R Core Team, 2022). R is a commonly used statistical programming language that is open source and freely available (Mann, 2020).

Statistical analyses for this research will include two primary phases. The first phase is to test the hypothesis that there is an identifiable emotional pattern associated with various stages of therapy as it pertains to patient outcome (i.e., discontinuing sessions, progress, continued suicidal ideation etc.). This hypothesis will be tested using outlier, trend, and anomaly detection methods. The specific method of choice is dependent upon data distribution (Aggarwal, 2016). Initially, we will be investigating the data for similarity in

emotional patterns for subjects that experienced the same or similar therapy outcome. Therapy outcome will serve as a labeled data set used to test predictive validity.

The second phase is to test predictive validity. We will apply multiple unsupervised machine learning classification algorithms to a partitioned dataset for model construction. These include random forest, naive bayes, logistic regression, and support vector machine (SVM) algorithms. The models will be optimized using this data (training set). Predictive validity will be determined by applying the highest performing model, as determined by accuracy, precision, recall, and F1 scores on the training set, to a holdout data set (test set) and inspection of the same key performance metrics. This process will be iteratively applied to each category of labeled data.

7. Bibliography

Aggarwal, C. C. (2016). Outlier analysis (2nd ed.). Springer.

Barrett, L. F., & Salovey, P. (Eds.). (2002). The wisdom in feeling: Processes underlying emotional intelligence. New York: Guilford.

Becker-Asano, C., & Wachsmuth, I. (2008). Affect simulation with primary and secondary emotions. Intelligent Virtual Agents, 15-28. https://doi.org/10.1007/978-3-540-85483-8_2

Brown, G. K., Ten Have, T., Henriques, G. R., Xie, S. X., Hollander, J. E., & Beck, A. T. (2005). Cognitive therapy for the prevention of suicide attempts: a randomized controlled trial. Jama, 294(5), 563-570.

Bryan, C. J., Clemans, T. A., Hernandez, A. M., Mintz, J., Peterson, A. L., Yarvis, J. S., ... & STRONG STAR Consortium. (2016). Evaluating potential iatrogenic suicide risk in trauma-focused group cognitive behavioral therapy for the treatment of PTSD in active duty military personnel. Depression and anxiety, 33(6), 549-557.Chancellor, S., & De Choudhury, M. (2020). Methods in predictive techniques for mental health status on social media: A critical review. *Digital Medicine, 3*(1). https://doi.org/10.1038/s41746-020-0233-7

De Choudhury, M., Gamon, M., Counts, S., & Horvitz, E. (2021). Predicting depression via social media. Proceedings of the International AAAI Conference on Web and Social Media, 7(1), 128-137. https://doi.org/10.1609/icwsm.v7i1.14432

Ekman, P., Friesen, W. V., & Tomkins, S. S. (1971). Facial affect scoring technique: A first validity study. Semiotica, 3(1), 37-58. https://doi.org/10.1515/semi.1971.3.1.37

Fontaine, J. J. R., & Scherer, K. R. (2013). Emotion is for doing: The Action tendency component. In J. J. R. Fontaine, K. R. Scherer, & C. Soriano (Eds.), Components of emotional meaning: A sourcebook (pp. 170–185). Oxford University Press.

Frijda, N. H. (1994). Emotions are functional, most of the time. In *The nature of emotion: Fundamental questions*. Oxford University Press.

Greenberg, L. S., Auszra, L., & Herrmann, I. R. (2007). The relationship among emotional productivity, emotional arousal and outcome in experiential therapy of depression. Psychotherapy Research, 17(4), 482-493. https://doi.org/10.1080/10503300600977800

Gual-Montolio, P., Jaén, I., Martínez-Borba, V., Castilla, D., & Suso-Ribera, C. (2022). Using artificial intelligence to enhance ongoing psychological interventions for emotional problems in real- or close to real-time: A systematic review. International Journal of Environmental Research and Public Health, 19(13), 7737. https://doi.org/10.3390/ijerph19137737

Guntuku, S. C., Yaden, D. B., Kern, M. L., Ungar, L. H., & Eichstaedt, J. C. (2017). Detecting depression and mental illness on social media: An integrative review. Current Opinion in Behavioral Sciences, 18, 43-49. https://doi.org/10.1016/j.cobeha.2017.07.005

Henry, K. E., Kornfield, R., Sridharan, A., Linton, R. C., Groh, C., Wang, T., Wu, A., Mutlu, B., & Saria, S. (2022). Human–machine teaming is key to AI adoption: Clinicians' experiences with a deployed machine learning system. Digital Medicine, 5(1). https://doi.org/10.1038/s41746-022-00597-7

Hedegaard, H., Curtin, S. C., & Warner, M. (2018). Suicide rates in the United States continue to increase. US Department of Health and Human Services, Centers for Disease Control and Prevention, National Center for Health Statistics.

Kauer, S. D., Mangan, C., & Sanci, L. (2014). Do online mental health services improve help-seeking for young people? A systematic review. Journal of Medical Internet Research, 16(3), e66.

Kusuma, K., Larsen, M., Quiroz, J. C., Gillies, M., Burnett, A., Qian, J., & Torok, M. (2022). The performance of machine learning models in predicting suicidal ideation, attempts, 12 and deaths: A meta-analysis and systematic review. Journal of Psychiatric Research, 155, 579-588. https://doi.org/10.1016/j.jpsychires.2022.09.050

Lane, R. D., Ryan, L., Nadel, L., & Greenberg, L. (2014). Memory reconsolidation, emotional arousal, and the process of change in psychotherapy: New insights from brain science. Behavioral and Brain Sciences, 38. https://doi.org/10.1017/s0140525x14000041

Lowe, R., & Ziemke, T. (2011). The feeling of action tendencies: On the emotional regulation of goal-directed behavior. Frontiers in Psychology, 2. https://doi.org/10.3389/fpsyg.2011.00346

Ma-Kellams, C., Or, F., Baek, J. H., & Kawachi, I. (2015). Rethinking suicide surveillance. Clinical Psychological Science, 4(3), 480-484. https://doi.org/10.1177/2167702615593475

Mann, R. E. (2020). Use of machine learning to predict ethical drift in law enforcement (9598) [Doctoral dissertation]. Walden Dissertations and Doctoral Studies.

Matsumoto, D., Frank, M. G., & Hwang, H. C. (2015). The role of intergroup emotions in political violence. Current Directions in Psychological Science, 24(5), 369-373. https://doi.org/10.1177/0963721415595023

Miner, A. S., Fleming, S. L., Haque, A., Fries, J. A., Althoff, T., Wilfley, D. E., Agras, W. S., Milstein, A., Hancock, J., Asch, S. M., Stirman, S. W., Arnow, B. A., & Shah, N. H.

(2022). A computational approach to measure the linguistic characteristics of psychotherapy timing, responsiveness, and consistency. Mental Health Research, 1(1). https://doi.org/10.1038/s44184-022-00020-9

Ophir, Y., Tikochinski, R., Asterhan, C., Sisso, I., & Reichart, R. (2020). Deep neural networks detect suicide risk from textual Facebook posts. Scientific Reports, 10(1). https://doi.org/10.31234/osf.io/k47hr

Osenbach, J. E., O'Brien, K. M., Mishkind, M., & Smolenski, D. J. (2013). Synchronous telehealth technologies in psychotherapy for depression: A meta-analysis. Depression and Anxiety, 30(11), 1058-1067.

R Core Team (2022). R: A language and environment for statistical computing. R Foundation for Statistical Computing, Vienna, Austria. URL https://www.R-project.org/

Resick, P. A., Wachen, J. S., Dondanville, K. A., Pruiksma, K. E., Yarvis, J. S., Peterson, A. L., ... & Litz, B. T. (2017). Effect of group vs individual cognitive processing therapy in active-duty military seeking treatment for posttraumatic stress disorder: A randomized clinical trial. JAMA psychiatry, 74(1), 28-36.

Rudd, M. D., Bryan, C. J., Wertenberger, E. G., Peterson, A. L., Young-McCaughan, S., Mintz, J., ... & Wilkinson, E. (2015). Brief cognitive-behavioral therapy effects on post-treatment suicide attempts in a military sample: results of a randomized clinical trial with 2-year follow-up. American journal of psychiatry, 172(5), 441-449.

Saeed, S. A., Diamond, J., & Bloch, R. M. (2011). Use of telepsychiatry to improve care for people with mental illness in rural North Carolina. NC Med J, 72(3), 219-222.

Sinyor, M., Williams, M., Mitchell, R., Zaheer, R., Bryan, C. J., Schaffer, A., ... & Selchen, S. (2020). Cognitive behavioral therapy for suicide prevention in youth admitted to hospital following an episode of self-harm: a pilot randomized controlled trial. Journal of affective disorders, 266, 686-694.

Sloan, D. M., Gallagher, M. W., Feinstein, B. A., Lee, D. J., & Pruneau, G. M. (2011). Efficacy of telehealth treatments for posttraumatic stress-related symptoms: A meta-analysis. Cognitive Behaviour Therapy, 40(2), 111-125.

Sloan, D. M., & Kring, A. M. (2007). Measuring changes in emotion during psychotherapy: Conceptual and methodological issues. Clinical Psychology: Science and Practice, 14(4), 307-322. https://doi.org/10.1111/j.1468-2850.2007.00092.x

Stanley, I. H., Hom, M. A., & Joiner, T. E. (2015). Mental health service use among adults with suicide ideation, plans, or attempts: results from a national survey. Psychiatric services, 66(12), 1296-1302.

Tanana, M. J., Soma, C. S., Kuo, P. B., Bertagnolli, N. M., Dembe, A., Pace, B. T., Srikumar, V., Atkins, D. C., & Imel, Z. E. (2021). How do you feel? Using natural language processing to automatically rate emotion in psychotherapy. Behavior Research Methods, 53(5), 2069-2082. https://doi.org/10.3758/s13428-020-01531-z

Tarrier, N., Taylor, K., & Gooding, P. (2008). Cognitive-behavioral interventions to reduce suicide behavior: a systematic review and meta-analysis. Behavior modification, 32(1), 77-108.

Taubitz, F., Büdenbender, B., & Alpers, G. W. (2022). What the future holds: Machine learning to predict success in psychotherapy. Behaviour Research and Therapy, 156, 104116. https://doi.org/10.1016/j.brat.2022.104116

Walsh, C. G., Ribeiro, J. D., & Franklin, J. C. (2017). Predicting risk of suicide attempts over time through machine learning. Clinical Psychological Science, 5(3), 457-469. https://doi.org/10.1177/2167702617691560

Zhang, T., Schoene, A. M., Ji, S., & Ananiadou, S. (2022). Natural language processing applied to mental illness detection: A narrative review. Digital Medicine, 5(1). https://doi.org/10.1038/s41746-022-00589-7
