# Supplementary material for: Telehealth Brief Cognitive Behavioral Therapy for Suicide Prevention: A Randomized Clinical Trial
Source: JAMA Netw Open. 2024 Nov 12;7(11):e2445913. doi: 10.1001/jamanetworkopen.2024.45913 (PMC11558477; doi:10.1001/jamanetworkopen.2024.45913)
Supplement: Supplement 2. — Data Sharing Statement [file jamanetwopen-e2445913-s002.pdf]

Data Sharing Statement

Baker. Telehealth Brief Cognitive Behavioral Therapy for Suicide Prevention. *JAMA Netw Open*. Published November 12, 2024. doi:10.1001/jamanetworkopen.2024.45913

Data
Additional Information: www.clinicaltrials.gov, NCT04844294, https://clinicaltrials.gov/study/NCT04844294?id=NCT04844294&rank=1
Data available: Yes
Data types: Deidentified participant data
How to access data: The data that the support the findings of this study are available from the authors upon reasonable request. Please email craig.bryan@osumc.edu for inquiries related to accessing deidentified participant data.
When available: With publication

Supporting Documents
Document types: None

Additional Information
Who can access the data: All de-identified participant data collected as part of this study will be available as raw individual-level data for sharing with external researchers working at an institution with a Federal wide Assurance (FWA) for the Protection of Human Subjects.
Types of analyses: When data are shared, no limits will be placed on how the data are used, although recipients must agree to not transfer the data to other users and to use the data for research purposes only. Co-authorship will not be required for data sharing.
Mechanisms of data availability: Data will be shared with investigator support facilitated via a signed data use agreement.
